# The Humbug of Closing Beds

**Published:** 1914-03-21

**Authors:** 


### THE HUMBUG OF CLOSING BEDS.

We publish elsewhere in this issue a full report of an address given by Sir Henry Burdett, when presiding over the anniversary meeting of the Leicester Royal Infirmary last week. The speech, which covers a very wide ground, is far from being merely of local interest, and there is one passage especially to which we wish to draw attention here. Dealing with the financial difficulties complained of at the neighbouring county hospital of Loughborough, the speaker said that the only course suggested by it, if the money were not raised, was to close the beds. He then proceeded to show the fallacy at the root of this suggestion, and characterised the closing of beds as a humbug. We particularly commend that paragraph of the speech which shares the title at the head of this article to the consideration of our readers, for while the principle is of universal application it gains point and trenchancy from the particular illustration which occasioned it.

As we do not feel able to improve the statements by which the policy of closing beds is shown to be a humbug, to which only incapable administrators could resort, we will not attempt to restate the arguments here. But an occasion must not be lost for driving home this fact: that if a simple test were required to judge the administrative capacity of voluntary hospital managers at a given time, no more suggestive one could be found than the proportion of institutions uttering this foolish threat of "closing beds." Luckily the proportion would always be small and the number implicated few. Nevertheless, the general high standard of voluntary hospital management is lowered by a handful of backsliding institutions, who endeavour to conceal their administrative bankruptcy and want of initiative in raising funds by regularly saying that beds must be closed, and occasionally also by closing them. This closing has never proved a solution, and, as might be expected from a policy which is the outcome of ineptitude, has frequently led to the cry that the whole hospital must be closed. This fact is enough to expose the humbug, and we hope that the committees of voluntary hospitals everywhere will take note of it, so that it shall be felt a public confession of incompetency on the part of any board of management to utter or endorse any suggestion of the kind. The really expensive part of hospital management is the central administration, and as this must remain practically constant whether a ward more or a ward less be in use, you cannot save out of your total expenditure the cost per bed of each you decide to close. The remedy for failing funds is an enthusiastic administrator, of the type with which the voluntary system has made us familiar, and it is noteworthy that wherever such a man has appeared the suggestion of closing beds is never made or thought of. Not Loughborough only, but Worcester Infirmary and certain other offenders in this respect, should take this truth to heart, and realise that the threat to close beds is the infallible outward sign of incompetent hospital managers.

